# The impact of COVID-19 on the treatment of opioid use disorder in carceral facilities: a cross-sectional study

**DOI:** 10.1186/s40352-022-00199-1

**Published:** 2022-12-19

**Authors:** Elizabeth C. Saunders, Milan F. Satcher, Laura B. Monico, Ryan D. McDonald, Sandra A. Springer, David Farabee, Jan Gryczynski, Amesika Nyaku, Donald Reeves, Lynn E. Kunkel, Alysse M. Schultheis, Robert P. Schwartz, Joshua D. Lee, Lisa A. Marsch, Elizabeth Needham Waddell

**Affiliations:** 1 Center for Technology and Behavioral Health, Geisel School of Medicine at Dartmouth College, 46 Centerra Parkway, Suite 315, Lebanon, NH 03766 USA; 2 Department of Community and Family Medicine, Dartmouth-Hitchcock Medical Center, Lebanon, NH USA; 3 Friends Research Institute, Baltimore, MD USA; 4 New York University Grossman School of Medicine, New York, NY USA; 5 Department of Internal Medicine, Section of Infectious Diseases, Yale School of Medicine, New Haven, CT USA; 6 Division of Infectious Diseases, Rutgers New Jersey Medical School, New Brunswick, NJ USA; 7 Rutgers University Correctional Health Care, Rutgers-Robert Wood Johnson Medical School, Trenton, NJ USA; 8 Oregon Health and Science University –Portland State University School of Public Health and Addiction Medicine Section, Division of General Internal Medicine & Geriatrics, Oregon Health and Science University, Portland, OR USA

**Keywords:** Medication for opioid use disorder (MOUD), Opioid use disorder, Buprenorphine, Naltrexone, COVID-19, Carceral settings

## Abstract

While the COVID-19 pandemic disrupted healthcare delivery everywhere, persons with carceral system involvement and opioid use disorder (OUD) were disproportionately impacted and vulnerable to severe COVID-associated illness. Carceral settings and community treatment programs (CTPs) rapidly developed protocols to sustain healthcare delivery while reducing risk of COVID-19 transmission. This survey study assessed changes to OUD treatment, telemedicine use, and re-entry support services among carceral and CTPs participating in the National Institute on Drug Abuse (NIDA)-funded study, Long-Acting Buprenorphine vs. Naltrexone Opioid Treatments in Criminal Justice System-Involved Adults (EXIT-CJS) study. In December 2020, carceral sites (*n* = 6; median pre-COVID 2020 monthly census = 3468 people) and CTPs (*n* = 7; median pre-COVID 2020 monthly census = 550 patients) participating in EXIT-CJS completed a cross-sectional web-based survey. The survey assessed changes pre- (January–March 2020) and post- (April–September 2020) COVID-19 in OUD treatment, telemedicine use, re-entry supports and referral practices. Compared to January–March 2020, half of carceral sites (*n* = 3) increased the total number of persons initiating medication for opioid use disorder (MOUD) from April–September 2020, while a third (*n* = 2) decreased the number of persons initiated. Most CTPs (*n* = 4) reported a decrease in the number of new admissions from April–September 2020, with two programs stopping or pausing MOUD programs due to COVID-19. All carceral sites with pre-COVID telemedicine use (*n* = 5) increased or maintained telemedicine use, and all CTPs providing MOUD (*n* = 6) increased telemedicine use. While expansion of telemedicine services supported MOUD service delivery, the majority of sites experienced challenges providing community support post-release, including referrals to housing, employment, and transportation services. During the COVID-19 pandemic, this small sample of carceral and CTP sites innovated to continue delivery of treatment for OUD. Expansion of telemedicine services was critical to support MOUD service delivery. Despite these innovations, sites experienced challenges providing reintegration supports for persons in the community. Pre-COVID strategies for identifying and engaging individuals while incarcerated may be less effective since the pandemic. In addition to expanding research on the most effective telemedicine practices for carceral settings, research exploring strategies to expand housing and employment support during reintegration are critical.

**Supplementary Information:**

The online version contains supplementary material available at 10.1186/s40352-022-00199-1.

## Introduction

Prior to the COVID-19 pandemic, the United States (US) faced an ethical and public health crisis of mass incarceration after decades-long reliance on criminalization for social regulation. As of February 2020, the US represented less than 5% of the global general population but actively incarcerated 2.3 million people, or 20% of the global incarcerated population (Sawyer & Wagner, 2020; Wagner & Bertram, 2020). Persons who are incarcerated in the US are disproportionately impoverished, minoritized, and experience a higher burden of chronic comorbidities and premature death, compared to the general population (Kajeepeta et al., 2021; Rabuy & Kopf, 2015; Wildeman & Wang, 2017).

At the intersection of US drug policy and the opioid epidemic, an estimated 65% of people who are incarcerated have an active substance use disorder (National Institute on Drug Abuse (NIDA), National institutes of Health, & U.S. Department of Health and Human Services, 2020). During community re-entry, persons with recent incarceration experience a high rate of return to opioid use and a 129-times greater relative risk of fatal drug overdose in their first 2 weeks of re-entry compared to the general population (Binswanger et al., 2007; Binswanger et al., 2012; Binswanger et al., 2013). Clinical research has found that medications for opioid use disorder (MOUD) can effectively reduce opioid-related health disparities (e.g., return to substance use, fatal overdose, infectious disease, discontinuity of care, re-incarceration) among persons with recent incarceration (Brinkley-Rubinstein et al., 2018; Evans et al., 2022; Green et al., 2018; Lee et al., 2016; Lee et al., 2021; Rich et al., 2015; Springer et al., 2012; Springer et al., 2018). In recent years, this research has led to expansion of opioid use disorder (OUD) treatment in many carceral systems and the strengthening of OUD care transitions between carceral and community care sites (The Pew Charitable Trusts, 2020).

However, the onset of the COVID-19 pandemic in March 2020 caused mass disruption to health care delivery and clinical research as standard clinical practices rapidly changed to mitigate viral transmission. Health care mitigation strategies included reduced volume of in-person visits, delayed provision of elective and non-acute services, increased use of infection control practices in physical care settings (e.g., hand washing, physical distancing, personal protective equipment, case isolation), and increased utilization of remotely delivered care (e.g., telemedicine) (Kuy et al., 2020; Stuart, 2020). In March 2020, outpatient providers across specialties saw only 40% of the typical pre-pandemic volume for in-person visits amidst clinical practice changes and patient avoidance of health care environments (Czeisler et al., 2020; Mehrotra et al., 2021). Similarly, clinical trials were disrupted and delayed due to modified operations instituted by clinical care partners and research oversight committees (McDermott & Newman, 2021).

Persons who are incarcerated are highly vulnerable to acquiring COVID-19 and experiencing severe outcomes from the infection. Many of the most severe COVID-19 outbreaks in the US have occurred in carceral settings, where the risk of acquiring a COVID-19 infection is more than four-fold higher and the rate of COVID-19-associated death is more than three-fold higher than the non-incarcerated population (Mukherjee & El-Bassel, 2020; Schnepel et al., 2021). COVID-19’s transmission and severity in carceral settings may be mediated by limited spatial autonomy, overcrowded congregate living environments, hygiene restrictions, frequent exposure to community contacts (e.g., incoming residents, staff, site visitors), poor baseline health status, and living conditions that undermine immune functioning (e.g., chronic stress, social isolation, poor nutrition, persistent sleep disruption) (Chandra, 1997; Cook et al., 2015; Morris et al., 2021; Mukherjee & El-Bassel, 2020; Pressman et al., 2005; Sawyer, 2017; Segerstrom & Miller, 2004; Wurcel et al., 2020; Zee & Turek, 2006). Persons with a history of OUD have additional risks for COVID-19, with 2.4 times higher likelihood of acquiring COVID-19 and 1.4 times higher mortality from the infection than persons without OUD (Baillargeon et al., 2021; Wang et al., 2020). These risks may be mediated by immunosuppressive effects of chronic substance use, cardiopulmonary compromise from opioid-related respiratory depression and concomitant COVID-19 infection, and frequent interpersonal contacts necessary to sustain active substance use (Cowan et al., 2021; Volkow, 2020). The disparities in COVID-19 outcomes among populations vulnerable to incarceration called for strategies to prevent COVID-19 transmission in carceral settings while accounting for the risk of disrupting OUD care and exacerbating opioid-related health disparities, particularly during re-entry (Howell et al., 2020; Mukherjee & El-Bassel, 2020).

The current cross-sectional survey study assessed the health care mitigation strategies and OUD treatment changes adopted in response to the COVID-19 pandemic among carceral and community treatment program (CTP) sites participating in the National Institute on Drug Abuse (NIDA)-funded Long-Acting Buprenorphine vs. Naltrexone Opioid Treatments in Criminal Justice System-Involved Adults (EXIT-CJS) study (Waddell et al., 2021). The purpose of this survey was to examine the impact of COVID-19 on OUD treatment, telemedicine services, re-entry support and referral practices among participating carceral and CTP sites.

## Methods

### Study design and procedures

In December 2020, carceral staff (*n* = 6) and CTP staff (*n* = 7) participating in the EXIT-CJS study completed a cross-sectional web-based survey. Carceral and CTP sites were located in New Jersey, New York, Delaware, Oregon, Connecticut, and New Hampshire (Waddell et al., 2021). Information about the survey and the survey link were emailed to the primary EXIT-CJS contact(s) at each site by the EXIT-CJS research team. Participation was voluntary, consent obtained through the web-based survey platform, and all EXIT-CJS sites participated. The survey was reviewed and approved by the New York University School of Medicine Institutional Review Board.

### Survey

Staff at carceral and CTP sites completed a brief (15–30 minute) web-based survey developed for this study. The survey content was developed after reviewing the published literature on changes to OUD treatment during the COVID-19 pandemic, reviewing items developed for the Justice Community Opioid Innovation Network (JCOIN) jail survey (Ducharme et al., 2021; Scott & Dennis, 2019), and consulting with experts involved in OUD treatment and the EXIT-CJS study. One survey was developed for carceral sites (Supplement 1; 55 items), and a second survey was developed for CTP sites (Supplement 2; 42 items). The survey assessed changes pre- (January–March 2020) and post- (April–September 2020) COVID-19 onset in the total census of the facility, system or treatment program, the presence of OUD screening and treatment, the type of OUD treatments offered, the presence and utilization of telemedicine care, re-entry support for housing, transportation, employment, and referral practices for OUD treatment post-release. Additionally, the survey gathered information on the implementation of COVID-19 mitigation strategies.

### Respondents

One respondent completed the survey at each carceral and CTP site. Carceral site respondent roles included Health Services Leadership (*n* = 3), Security/Operations Leadership (*n* = 1), Planning/Research/Re-entry staff (*n* = 1), or Site Investigators/Corrections Manager (*n* = 1). Three carceral facility respondents also engaged in the provision of direct medical care. CTP respondent roles included CEO/Medical Director (*n* = 2), Administrator (*n* = 2), Clinical Care Provider/Prescriber (*n* = 2), or Research Associate (*n* = 1). Four CTP respondents were also treatment providers within their organization.

### Analysis

Survey data were collected and managed using the secure REDCap system hosted at New York University (Harris et al., 2019). Rates of missing data were calculated by item and overall. Descriptive statistics were calculated using Stata, Version 14.0 (StataCorp., 2015).

## Results

### Site characteristics

Carceral and CTP sites varied in their system structure and program census. Carceral sites (Table 1) consisted of local jails (*n* = 2), state prisons (*n* = 2), and unified corrections systems (*n* = 2). While the two jail sites were single facilities, the other carceral sites included multiple facilities (median: 9 facilities, range: 4–13 facilities). Across the carceral sites, the median monthly pre-COVID 2020 census was 3467 people (range: 401–18,000 people). All carceral sites (*n* = 6) prescribed MOUD. While sublingual buprenorphine (*n* = 6) and extended-release naltrexone (*n* = 6) were available at all carceral sites pre-COVID, methadone (*n* = 5), sublingual buprenorphine/naloxone (*n* = 4) and extended-release buprenorphine (*n* = 3) were also available in at least half of the sites pre-COVID.

**Table 1:** Organization and opioid use disorder (OUD) treatment characteristics of participating carceral system sites (*n* = 6)

	Carceral sites (*n* = 6)
**Organization characteristics,** n (%)	
**Corrections system structure**	
Unified corrections system	2 (33.3%)
State prison	2 (33.3%)
Local jail	2 (33.3%)
**Single facility versus organization/system**	
Single facility	2 (33.3%)
Organization/system	4 (66.7%)
Number of facilities, median (range)	9 (4–13) facilities
**Health care delivery model**	
Contracted model	4 (66.7%)
Hybrid model	1 (16.7%)
Direct services model	0 (0%)
Other^a^	1 (16.7%)
**Opioid use disorder (MOUD) treatment characteristics,** n (%)	
**Organization/facility prescribes MOUD**	6 (100%)
**MOUD prescribed for:**	
Opioid withdrawal management	6 (100%)
Maintenance for any persons with OUD	6 (100%)
Induction and maintenance only immediately prior to release	6 (100%)
Maintenance only for pregnant women	3 (50.0%)
Maintenance only for persons entering on MOUD	4 (66.7%)
**Medications available within organization/facility**	
Methadone	5 (83.3%)
Sublingual buprenorphine	6 (100%)
Extended-release buprenorphine	3 (50.0%)
Sublingual buprenorphine/naloxone	4 (66.7%)
Extended-release naltrexone	6 (100%)
**Available non-medication treatment for SUDs**	
Peer mentor/navigator/recovery coach	5 (83.3%)
Outpatient substance use treatment	4 (66.7%)
Mutual- or self-help group meetings	4 (66.7%)
Transfer to residential or inpatient substance use treatment in the community	4 (66.7%)
Intensive outpatient substance use treatment	3 (50.0%)
Co-occurring substance use and mental health services	3 (50.0%)
Onsite short- or long-term residential treatment	3 (50.0%)
Other recovery-based unit	3 (50.0%)
Therapeutic community	2 (33.3%)
**Telemedicine use,** n (%)	
**Prior to COVID-19, use of telemedicine for:**	
Treatment of any health condition	5 (83.3%)
Inducting incarcerated/detained persons on MOUD	1 (20.0%)
**Use of technology/methods used by persons who are incarcerated/detained to participate in substance use treatment or recovery support**	
Video calls	5 (83.3%)
Tablets	4 (66.7%)
Computers	3 (50.0%)
Kiosks	1 (16.7%)
Portable kiosks	1 (16.7%)
Cell phones	1 (16.7%)
Other applications or software programs	1 (16.7%)
Internet	0 (0%)
Text messaging	0 (0%)
Email	0 (0%)
**Re-entry support and post-release referral practices,** n (%)	
**In the month prior to release, staff in the facility/organization engage in:**	
Scheduling appointments with MOUD providers in the community	6 (100%)
Providing assistance completing intake paperwork for MOUD providers in the community	6 (100%)
Facilitating exchange of key information with MOUD providers in the community	6 (100%)
Providing incarcerated/detained persons with the name(s) of MOUD providers in the community	6 (100%)
Assisting with reactivation and/or applying for Medicaid, Veterans benefits, or other types of insurance for payment of MOUD	6 (100%)
Inducting incarcerated/detained persons on MOUD	5 (83.3%)
Coordinating MOUD services with probation/parole	5 (83.3%)
Connecting incarcerated/detained persons to a peer mentor/navigator/recovery coach	5 (83.3%)
Providing a bridge supply of MOUD	5 (83.3%)
Providing a written prescription for MOUD	4 (66.7%)
Providing or arranging transportation to a MOUD provider in the community	2 (33.3%)
**Facility/organization provides naloxone kits at release**	6 (100%)
**Naloxone kits at release provided to:**	
Everyone	1 (16.7%)
Only individuals with opioid use problems/OUD	4 (66.7%)
Individuals with any substance use disorder	1 (16.7%)

^a^ Medication for opioid use disorder services are contracted, but medical providers are Department of Corrections employees

CTPs (Table 2) consisted of opioid treatment programs (*n* = 2), stand-alone outpatient medical or behavioral health centers (*n* = 2), tertiary care medical centers (*n* = 1), state health departments (*n* = 1) and other (*n* = 1). Four CTPs had contracts or treatment tracks specifically designed for adults with OUD involved with the carceral system. Most CTPs also prescribed MOUD (*n* = 6), including extended-release naltrexone (*n* = 6), oral naltrexone (*n* = 5), sublingual buprenorphine/naloxone (*n* = 5), extended-release buprenorphine (*n* = 4), and methadone (*n* = 2) pre-COVID.

**Table 2:** Organization and opioid use disorder (OUD) treatment characteristics of participating community treatment program sites (*n* = 7)

	Community treatment program sites (*n* = 7)
**Organization characteristics,** n (%)	
**Type of organization**	
Opioid treatment program	2 (28.6%)
Stand-alone outpatient medical or behavioral health center	2 (28.6%)
Tertiary care medical center	1 (14.3%)
Health department	1 (14.3%)
Other	1 (14.3%)
**Contract or treatment track for justice-involved adults with OUD**	4 (57.1%)
**Opioid use disorder (MOUD) treatment characteristics,** n (%)	
**MOUD prescribed for:**	
Opioid withdrawal management	5 (71.4%)
Maintenance for adults with OUD	5 (71.4%)
Maintenance for pregnant women	4 (57.1%)
Maintenance for juveniles	1 (14.3%)
**Medications available within organization/facility**	
Methadone	2 (28.6%)
Sublingual buprenorphine	5 (71.4%)
Extended-release buprenorphine	4 (57.1%)
Sublingual buprenorphine/naloxone	5 (71.4%)
Extended-release naltrexone	6 (85.7%)
Oral naltrexone	5 (71.4%)
**Available non-medication treatment for SUDs offered to carceral-involved patients**	
Outpatient substance use treatment	5 (71.4%)
Co-occurring substance use and mental health services	5 (71.4%)
Intensive outpatient treatment	4 (57.1%)
Peer mentor/navigator/recovery coach	4 (57.1%)
Mutual- or self-help group meetings	2 (28.6%)
Transfer to residential treatment/inpatient treatment	2 (28.6%)
Other: Opioid treatment program	1 (14.3%)
**Telemedicine use,** n (%)	
**Prior to COVID-19, use of telemedicine for:**	
Treatment of any health condition	2 (33.3%)
Inducting patients on MOUD	1 (16.7%)
Maintaining patients on MOUD	2 (33.3%)
**Use of technology/methods used by patients with OUD to participate in substance use treatment or recovery support**	
Video calls	6 (85.7%)
Computers	6 (85.7%)
Cell phones	6 (85.7%)
Tablets	5 (71.4%)
Text messaging	5 (71.4%)
Email	4 (57.1%)
Internet	4 (57.1%)
Other applications or software programs: Zoom and reSET	1 (14.3%)
Kiosks	1 (14.3%)
Portable kiosks	0 (0%)
**Wraparound services and other supports,** n (%)	
**Program provides naloxone kits**	7 (100%)
**Naloxone kits provided to:**	
Everyone	2 (28.6%)
Only individuals with opioid use problems/OUD	3 (42.9%)
Other: Offered to all OUD patients; Kit/prescription provided to anyone who asks We have them on hand to administer in the office if needed	2 (28.6%)

### Implementation of COVID-19 harm reduction strategies or policies

Five carceral and seven CTP sites reported implementing COVID-19 mitigation policies. Carceral sites most commonly implemented change in movement policies (*n* = 5) and reduced face-to-face visitation of incarcerated/detained persons (*n* = 4), jail/prison entries/admissions (*n* = 3), and face-to-face medical visits (*n* = 2). COVID-19 mitigation strategies were also implemented within carceral MOUD programs. Among the five sites providing methadone, two altered methadone dosing procedures. These changes included providing cloth masks to patients (*n* = 2), implementing enhanced cleaning regimens (*n* = 2), screening patients for COVID-19 symptoms prior to dosing (*n* = 2), and eliminating/pausing the offsite transport of patients for external methadone dosing (*n* = 1). Only one carceral site reported changing buprenorphine dosing procedures post-COVID by providing cloth masks to patients (*n* = 1), enhancing cleaning regimens (*n* = 1), and screening patients for COVID-19 symptoms prior to dosing (*n* = 1).

All CTPs enacted policies to reduce the risk of COVID-19 transmission. Six CTPs reduced face-to-face medical visits, two limited new admissions, and one eliminated medical co-pays. Two CTPs implemented COVID-19 symptom screening, mask/face covering requirements and work from home protocols, and one CTP stopped providing transportation to appointments. Two CTPs providing methadone implemented enhanced cleaning regimens (*n* = 2), altered dosing locations (*n* = 2), the provision of cloth masks (*n* = 2), and COVID-19 symptom screening policies (*n* = 2). Three of the six CTPs offering buprenorphine also reduced the number of in-person visits for dosing (*n* = 3), screened patients for COVID-19 symptoms (*n* = 1), enhanced cleaning regimens (*n* = 1), altered dosing locations (*n* = 1), and provided cloth masks to patients (*n* = 1).

### Changes to MOUD treatment

Compared to January–March 2020, half (*n* = 3) of carceral sites reported decreases in the number of persons screened for OUD from April–September 2020 (Table 3). Overall, half of carceral sites (*n* = 3) increased the total number of persons being inducted on MOUD during incarceration from April–September 2020, while a third (*n* = 2) decreased the total number of persons being inducted on MOUD during incarceration. Three carceral sites reported decreases in the number of incarcerated/detained persons receiving sublingual buprenorphine and two sites reported decreases in the numbers of persons receiving extended-release buprenorphine. Most carceral sites experienced no changes in the number of persons receiving MOUD at release.

**Table 3:** Changes to treatment for opioid use disorder, telemedicine services, referral and release planning among EXIT-CJS carceral sites (*n* = 6) during the post-COVID-19 period (April–September 2020)

	**Increased**	**No noticeable change**	**Decreased**
	n (%)		
**Treatment for opioid use disorder,** n (%)			
**Before and after COVID-19, has your facility/organization had changes in the:**			
Number of persons screened for opioid use disorder	0 (0%)	3 (50.0%)	3 (50.0%)
**Since COVID-19, has your facility has any changes in the:**			
Number of providers waivered to prescribe buprenorphine	1 (16.7%)	5 (83.3%)	0 (0%)
Demand for MOUD	1 (16.7%)	4 (66.7%)	1 (16.7%)
**Of those receiving MOUD for maintenance, how did the number of persons receiving methadone, buprenorphine, or naltrexone change in the months before and after COVID-19?**			
Methadone^a^	1 (25.0%)	3 (75.0%)	0 (0%)
Sublingual buprenorphine or buprenorphine/naloxone^a^	2 (33.3%)	1 (16.7%)	3 (50.0%)
Extended-release buprenorphine^a^	1 (33.3%)	0 (0%)	2 (66.7%)
Extended-release naltrexone^a^	0 (0%)	3 (75.0%)	1 (25.0%)
**How did the number of incarcerated/detained persons entering on MOUD, continued on MOUD, and tapered off MOUD changed within your facility/organization?**			
Entering facility on MOUD	1 (16.7%)	4 (66.7%)	1 (16.7%)
Continued on MOUD	1 (16.7%)	5 (83.3%)	0 (0%)
Tapered off MOUD prior to release	0 (0%)	5 (83.3%)	1 (16.7%)
**How did the number of incarcerated/detained persons being inducted onto MOUD in your facility/organization change?**			
Total number of persons inducted on MOUD	3 (50.0%)	1 (16.7%)	2 (33.3%)
Number started for withdrawal management only	1 (16.7%)	5 (83.3%)	0 (0%)
Number started for maintenance	2 (33.3%)	3 (50.0%)	1 (16.7%)
**In the months before and after COVID-19, did your organization have changes in the number of incarcerated/detained persons released:**			
On MOUD without a referral?	1 (16.7%)	4 (66.7%)	1 (16.7%)
Not on MOUD, but with a referral to community treatment for MOUD?	0 (0%)	5 (83.3%)	1 (16.7%)
On MOUD and referred to community treatment?	2 (33.3%)	3 (50.0%)	1 (16.7%)
**Changes to telemedicine use,** n (%)			
	**Increased**	**No noticeable change**	**Decreased**
**Since COVID-19, has your facility**^b^ **had any changes in use of telemedicine for:**			
All health conditions	4 (80.0%)	1 (20.0%)	0 (0%)
MOUD	3 (60.0%)	2 (40.0%)	0 (0%)
**Use of technology/methods by incarcerated/detained persons to participate in substance use treatment**			
Computers	3 (100%)	0 (0%)	0 (0%)
Tablets	3 (75.0%)	1 (25.0%)	0 (0%)
Portable kiosks	0 (0%)	1 (100%)	0 (0%)
Kiosks	0 (0%)	1 (100%)	0 (0%)
Cell phones	1 (100%)	0 (0%)	0 (0%)
Video calls	2 (50.0%)	2 (50.0%)	0 (0%)
Other applications or software programs	1 (100%)	0 (0%)	0 (0%)
**Changes to re-entry support and post-release referral practices,** n (%)			
	**Increased**	**No noticeable change**	**Decreased**
**In the month prior to release, how has COVID-19 impacted your facility/organization’s ability to:**			
Schedule appointments with MOUD providers in the community	1 (16.7%)	2 (33.3%)	3 (50.0%)
Provide assistance completing intake paperwork for MOUD providers in the community	1 (16.7%)	2 (33.3%)	3 (50.0%)
Facilitate exchange of key information with MOUD providers in the community	1 (16.7%)	4 (66.7%)	1 (16.7%)
Provide incarcerated/detained persons with the name(s) of MOUD providers in the community	2 (33.3%)	3 (50.0%)	1 (16.7%)
Coordinate MOUD services with probation/parole^c^	1 (20.0%)	3 (60.0%)	1 (20.0%)
Assist with reactivating and/or applying for Medicaid, Veterans benefits or other types of insurance for payment of MOUD	0 (0%)	6 (100%)	0 (0%)
Connect incarcerated/detained persons to a peer mentor/navigator/recovery coach?^c^	2 (40.0%)	2 (40.0%)	1 (20.0%)
Provide or arrange transportation to MOUD provider in the community	0 (0%)	2 (100%)	0 (0%)
Provide a bridge supply of MOUD^c^	0 (0%)	2 (100%)	0 (0%)
Provide a written prescription for MOUD^c^	1 (25.0%)	3 (75.0%)	0 (0%)
**Since COVID-19, how has your facility/organization’s provision of naloxone kits at release changed?**	2 (33.3%)	4 (66.7%)	0 (0%)
**Post-release, how has COVID-19 impacted your facility/organization’s ability to:**			
Locate housing for incarcerated/detained persons in the community	0 (0%)	2 (33.3%)	4 (66.7%)
Help incarcerated/detained persons find employment in the community	0 (0%)	2 (33.3%)	4 (66.7%)
Provide transportation home for incarcerated/detained persons	0 (0%)	5 (83.3%)	1 (16.7%)

^a^ Number of carceral sites providing: Methadone (*n* = 5), Sublingual buprenorphine or buprenorphine/naloxone (*n* = 6), Extended-release buprenorphine (*n* = 3), Extended-release naltrexone (*n* = 6); ^b^ Among 5 facilities providing telemedicine services pre-COVID; ^c^ Coordinate MOUD services (*n* = 5), Connect with peer (*n* = 5), Arrange transportation (*n* = 2), Provide bridge supply (*n* = 2), Provide written prescription (*n* = 4)

Across CTP sites, most CTPs (*n* = 4) decreased the overall number of persons admitted for OUD in April–September 2020 (Table 4), and two CTPs stopped or paused MOUD programs as a result of COVID-19. While the majority of CTPs reported no changes in the number of patients receiving extended-release buprenorphine and extended-release naltrexone, two sites experienced decreases in the number of persons receiving sublingual buprenorphine.

**Table 4:** Changes to treatment for opioid use disorder, telemedicine services, referral and release planning among EXIT-CJS community treatment program (CTP) sites (*n* = 7) during the post-COVID-19 period (April–September 2020)

	**Increased**	**No noticeable change**	**Decreased**
	n (%)		
**Treatment for opioid use disorder**^a^			
**Since COVID-19, have your clinical programs had any changes in the:**			
Number of persons admitted for OUD	2 (33.3%)	0 (0%)	4 (66.7%)
Percent of persons admitted for OUD versus other disorders	0 (0%)	4 (66.7%)	2 (33.3%)
Number of persons admitted needing opioid withdrawal management	0 (0%)	3 (60.0%)	2 (40.0%)
Number of admitted persons self-reporting or screening positive for fentanyl/fentanyl analogues	1 (2.0%)	4 (80.0%)	0 (0%)
**Since COVID-19, has your program has any changes in the:**			
Number of providers waivered to prescribe buprenorphine	1 (16.7%)	5 (83.3%)	0 (0%)
Demand for MOUD	3 (50.0%)	2 (33.3%)	1 (16.7%)
**Of those receiving MOUD for maintenance, how did the number of persons receiving methadone, buprenorphine, or naltrexone change in the months before and after COVID-19?**			
Methadone^b^	1 (25.0%)	2 (50.0%)	1 (25.0%)
Sublingual buprenorphine^b^	1 (16.7%)	3 (50.0%)	2 (33.3%)
Extended-release buprenorphine^b^	1 (16.7%)	4 (66.7%)	1 (16.7%)
Extended-release naltrexone^b^	0 (0%)	5 (83.3%)	1 (16.7%)
**In the months before and after COVID-19, how did the number of CJS-involved persons being inducted onto MOUD in your facility/organization change?**			
Total number of persons inducted on MOUD	1 (16.7%)	4 (66.7%)	1 (16.7%)
Number started for withdrawal management only	0 (0%)	6 (100%)	0 (0%)
Number started for maintenance	1 (16.7%)	4 (66.7%)	1 (16.7%)
**Changes to telemedicine use,** n (%)			
	**Increased**	**No noticeable change**	**Decreased**
**Since COVID-19, has your facility had any changes in use of telemedicine for:**^**a**^			
All health conditions	6 (100%)	0 (0%)	0 (0%)
MOUD	6 (100%)	0 (0%)	0 (0%)
**Since COVID-19, how has your facility/organization’s use of the following technology programs for substance use treatment or recovery support changed?**^b^			
Computers	6 (100%)	0 (0%)	0 (0%)
Tablets	5 (100%)	0 (0%)	0 (0%)
Kiosks	1 (100%)	0 (0%)	0 (0%)
Cell phones	5 (83.3%)	1 (16.7%)	0 (0%)
Internet	3 (75.0%)	1 (25.0%)	0 (0%)
Text messaging	4 (80.0%)	1 (20.0%)	0 (0%)
Email	3 (75.0%)	1 (25.0%)	0 (0%)
Video calls	6 (100%)	0 (0%)	0 (0%)
Other applications or software programs	0 (0%)	1 (100%)	0 (0%)
**Changes to wraparound services and other supports,** n (%)			
	**Increased**	**No noticeable change**	**Decreased**
**Since COVID-19, how has your facility/organization’s provision of naloxone kits at release changed?**	3 (42.9%)	4 (57.1%)	0 (0%)
**How has COVID-19 impacted your facility/organization’s ability to provide:**^c^			
Housing assistance and homeless supports	1 (16.7%)	0 (0%)	5 (83.3%)
Employment program and supports	0 (0%)	2 (33.3%)	4 (66.7%)
Transportation support	0 (0%)	2 (33.3%)	4 (66.7%)

^a^Among 6 CTPs providing MOUD treatment; ^b^ Number of CTPs providing: Methadone (*n* = 4), Sublingual buprenorphine (*n* = 6), Extended-release buprenorphine (*n* = 6), Extended-release naltrexone (*n* = 6); ^c^ Housing assistance, employment assistance, transportation support (*n* = 6)

### Changes to telemedicine services

All carceral sites with pre-COVID use of telemedicine (*n* = 5) either increased or maintained telemedicine use for both MOUD and other health conditions (Table 3). At least half of carceral sites providing telemedicine services through computers, tablets, and video calls increased use of these technologies. Similarly, all six CTPs providing MOUD increased telemedicine use post-COVID-19 (Table 4). CTPs increased use of computers, tablets, video calls, cell phones, and text messaging programs to provide treatment and recovery support.

### Changes to release planning, re-entry, and wraparound services

Compared to January–March 2020, most carceral sites reported no changes in April–September 2020 in facilitating key information exchanges with community MOUD providers (*n* = 4), coordinating MOUD services with probation and parole (*n* = 3), assisting with the reactivation of insurance for payment of MOUD (*n* = 6), and providing written (*n* = 3) or bridge prescriptions (*n* = 2) for MOUD (Table 3). Conversely, half of carceral sites reported a decreased ability to schedule appointments with MOUD providers in the community (*n* = 3) and to provide assistance completing paperwork for MOUD community providers (*n* = 3). Four of six carceral sites also endorsed a decreased ability to help incarcerated/detained persons locate housing and find employment post-release. The majority of CTPs likewise reported that COVID-19 decreased their program’s ability to provide housing assistance (*n* = 5), employment support (*n* = 4), and transportation supports (*n* = 4; Table 4).

## Discussion

In response to COVID-19-related disruptions in healthcare delivery and clinical research, this cross-sectional survey study assessed the healthcare mitigation strategies and changes to OUD treatment, telemedicine services, and re-entry supports adopted from April–September 2020 among carceral and CTP sites participating in the EXIT-CJS study. Although half of carceral sites reduced screening for OUD and decreased the numbers of persons receiving sublingual buprenorphine, most carceral sites operationally maintained MOUD delivery during the early months of the pandemic. COVID mitigation policies, including masking and COVID-19 symptom screening, were implemented in most carceral MOUD programs. These changes to carceral MOUD programs reported by EXIT-CJS study sites parallel changes occurring in other carceral programs during the early months of the COVID-19 pandemic (Bandara et al., 2020; Donelan et al., 2021; Duncan et al., 2021). Across the US, the mean monthly volume of MOUD at carceral sites increased throughout the pandemic, while MOUD volume at other residential healthcare programs remained relatively stable (Dadiomov et al., 2022). The increases observed at carceral sites were largely related to increased availability of buprenorphine/naloxone (Dadiomov et al., 2022). Despite challenges presented by the pandemic, legislation and recent court decisions supporting the provision of MOUD in carceral settings (Toyoshima et al., 2021) coupled with funding mechanisms, including State Opioid Response (SOR) grants (Substance Abuse and Mental Health Services Administration (SAMHSA), 2021) and enhanced telemedicine services, may have supported the ongoing provision of MOUD during the pandemic at EXIT-CJS sites and in other carceral settings (Dadiomov et al., 2022). Several carceral and CTP sites also increased the provision of extended-release buprenorphine and naltrexone. Expansion of long-acting MOUD formulations in carceral settings and the community could reduce the risk of COVID transmission by decreasing the number of visits required for dosing while providing effective MOUD treatment (Berk et al., 2021; Jarvis et al., 2018; Lee et al., 2018; Lee et al., 2021; Springer et al., 2018).

CTPs conversely reported a decrease in the number of new persons admitted with OUD, and two programs paused or stopped MOUD programs between April and September 2020. Results of a scoping review similarly found that community treatment programs for OUD often reduced their capacity early in the pandemic, though treatment availability generally improved later with increased utilization of telemedicine and take-home dosing (Kumar et al., 2022). Surveyed CTPs were located in six unique states. Considering the impact of local and state-level policies on CTPs’ ability to sustain MOUD treatment services during the pandemic is warranted. In March 2020, some US federal regulations on MOUD were revised on an emergency basis to facilitate access to take-home methadone or buprenorphine doses in opioid treatment programs (Substance Abuse and Mental Health Services Administration (SAMHSA), 2020), and increase telemedicine use for buprenorphine initiation (Drug Enforcement Administration, 2020b). Adoption of these regulations was inconsistent across states (Nesoff et al., 2021; Pessar et al., 2021). Though 44 states ultimately expanded access to MOUD through the Substance Abuse and Mental Health Services Administration (SAMHSA) Blanket Exception and the US Drug Enforcement Administration (DEA) buprenorphine waiver allowing for prescription of buprenorphine without an in-person evaluation (Pessar et al., 2021), 24 states had not formally implemented any federal policy for MOUD treatment expansion as of April 2020 (Nesoff et al., 2021). According to one estimate, the lack of state-level adoption of MOUD treatment expansion regulations could have resulted in up to half a million people without access to MOUD during the early months of the pandemic (Nesoff et al., 2021). Though the specific impact of MOUD disruption during COVID-19 on people with recent carceral system involvement is understudied, rising rates of opioid overdose deaths occurring through 2021 (National Center for Health Statistics, 2021) in the setting of a baseline disparate vulnerability to re-entry-related overdose prior to the pandemic (Binswanger et al., 2007) underscore the importance of implementing policies and practices to ensure continued access to MOUD during times of societal crisis. Examples of such proposed federal legislation include the Medicaid Reentry Act, which would permit all states to reactivate Medicaid coverage for incarcerated persons up to 30 days prior to release (117th Congress, 2021a). The Humane Correctional Health Care Act would repeal the Medicaid Inmate Exclusion Policy altogether and therefore more effectively safeguard against insurance gaps created by unpredictable jail and parole release dates (117th Congress, 2021b).

Both carceral and CTP sites innovated to continue delivery of treatment for OUD, including the expansion of telemedicine services. With federal regulations allowing telephone prescribing of buprenorphine for patients during the COVID-19 pandemic, including persons who are currently incarcerated (Drug Enforcement Administration, 2020a), sites had an unprecedented opportunity to expand telemedicine services for MOUD. Sites accelerated their use of computers, tablets, video calls, cell phones, and text messaging programs to provide MOUD treatment and other healthcare services. As individuals involved in the carceral system often face significant barriers to receiving treatment for substance use disorders (Belenko et al., 2013; Hunt et al., 2015), expansion of technology-based treatment (such as digital therapeutics) and telemedicine services for SUD may improve access to treatment for this population (Leach et al., 2021). Though a systematic review of telemedicine treatment in carceral settings suggested that patients generally express satisfaction with telepsychiatry services, satisfaction with MOUD services delivered via telemedicine in carceral settings should be explored (Cole et al., 2020; Tian et al., 2021). The effectiveness of telemedicine programs in carceral settings also varies (Leach et al., 2021; Tian et al., 2021). Research characterizing models of care utilizing telemedicine for MOUD and examining optimal models of technology-based OUD treatment in carceral settings is necessary to develop best-practice guidance and inform the implementation of telemedicine for MOUD.

While most carceral and CTP sites maintained the capacity to provide MOUD, their ability to provide community re-entry support declined. Both carceral and CTP sites reported challenges providing support for housing, transportation, and employment during reintegration into the community. In general, housing affordability and availability declined during the COVID-19 pandemic, amplifying housing inequalities (Jones & Grigsby-Toussaint, 2020; Rogers & Power, 2020). Coupled with economic challenges and job losses, community programs providing housing resources in the US experienced an increased demand for services (Pixley et al., 2021). In addition to demands on community services due to COVID-19, re-entry support systems may be overburdened due to moderately increased numbers of incarcerated persons being released into the community because of COVID-19 (Natoli et al., 2021; Prison Policy Initiative, 2022). Employment and housing are critical to successful reintegration into the community (Shinkfield & Graffam, 2009). Individuals lacking stable housing face higher risks for developing severe COVID-associated complications (Cha et al., 2021; Hsu et al., 2020; Montgomery et al., 2022), while stable housing is associated with improved treatment response and recovery outcomes (Pro et al., 2022; VanDeMark, 2007). Future research should explore policies and strategies to sustain and support comprehensive re-entry supports. As research shows that addressing dynamic risk factors including health, employment, housing, and social networks significantly impacts outcomes during community reintegration, policies providing increased funding and other resources for services addressing these domains are critical (Goger et al., 2021; Zhang et al., 2019).

Policies expanding funding and other resources for the development, evaluation and implementation of evidence-based re-entry supports for persons with OUD are also crucial. Emerging evidence supports the effectiveness of the Transitions Clinics Network model, which addresses the social determinants of health while providing OUD treatment (Howell et al., 2021). Peer navigation is another emerging method for providing support to individuals with OUD who are re-integrating into the community (Belenko et al., 2021; Bellamy et al., 2019; Tillson et al., 2022). Digital technology could also offer a novel method for providing or extending re-entry supports. Recent research suggests that persons with OUD are receptive to digital health interventions supporting engagement in MOUD (Langdon et al., 2022), and digital health tools providing additional re-entry support services may likewise hold promise.

Consistent with recommendations for reducing disease transmission (Natoli et al., 2021) and effective COVID-19 prevention strategies identified in a recent systematic review (Esposito et al., 2022), EXIT-CJS study sites implemented a variety of policies to reduce the spread of COVID-19, including change in movement policies, elimination of in-person visitations, and a reduction in face-to-face medical visits. These policies impacted models of care for individuals with OUD, and additionally affected ongoing research in both carceral and community settings. For researchers working with vulnerable populations with OUD during the COVID-19 pandemic, protecting participant safety through utilization of technology-based research methods is warranted. While implementing novel strategies to reduce the risk of COVID-19 transmission is critical for MOUD services delivery, MOUD research for persons involved in the carceral system will need to adapt to changes imparted by the COVID-19 pandemic. With expansion of new technologies to provide care for OUD in carceral and community settings, the same technologies may also support research on MOUD by facilitating virtual consents and research visits (Brezing et al., 2020). Virtual research visits conducted through HIPAA-compliant telehealth programs can allow researchers to safely meet with participants in both carceral and community settings (Brezing et al., 2020; Keesara et al., 2020). To safeguard participant privacy, researchers must consider how to ethically gather data about substance use behaviors and engagement in illegal activities during remote assessments (Englund et al., 2022). This may be particularly challenging in carceral or other residential settings where telephone or video communications may be monitored. While MOUD programs may reduce or eliminate requirements for urine drug screen testing as a result of COVID-19 (Pytell & Rastegar, 2021), MOUD research studies should consider novel approaches to obtaining this data. These might include mailing or delivering a take-home urine drug testing kit and either reviewing results by video or shipping kits to the research team. Alternatively, considering saliva testing or contracting with external laboratories may be useful (Brezing et al., 2020; Revoredo et al., 2021). Protocols for obtaining immediate assistance for participants in the circumstance of an overdose or mental health emergency are also imperative when conducting remote assessments (Englund et al., 2022). Study findings suggest that although some carceral settings may still have limited technological infrastructure (Jewkes & Reisdorf, 2016; Van De Steene & Knight, 2017), other carceral settings may increasingly have the technological infrastructure to support remote research activities. Further investment in improving technological infrastructure in carceral settings could support continued research activities and access to health services. Outside of carceral facilities, ensuring safe and equitable access to remote research activities is critical to avoid digital exclusion for participants in the community (Englund et al., 2022). Providing participants with devices and a secure, private place to access high-speed internet could facilitate the engagement and retention of individuals with OUD in the community.

Limitations to this study include the small number of programs surveyed and the focus on the early pandemic period. Only the studies participating in the EXIT-CJS study were surveyed. Aside from the small sample size and heterogeneous nature of the included sites, the carceral and CTP sites surveyed demonstrated their commitment to MOUD treatment research by agreeing to participate in the larger study and are likely not representative of the carceral or CTP systems at large. Comparing changes in OUD treatment and referral due to COVID-19 at EXIT-CJS sites to changes at facilities not involved in the study could provide useful information on how these changes vary across different carceral and community settings. In addition, the survey focused on changes occurring early in the pandemic, from April through September 2020. The study was conducted prior to the availability of vaccines for COVID-19. It is unclear how MOUD treatment, telemedicine use, or re-entry/wraparound support services changed after the early pandemic period, and with approval of vaccines. Finally, our survey assessed data on provision of services to individuals with known treatment need. We were not able to estimate changes in the proportion of adults in each setting with undiagnosed or undisclosed treatment need. Survey participants reported increases or declines in actual services delivered and actual referrals to clinical services. In particular, local jails observed qualitative changes in the population housed during the pandemic due to reduced housing capacity and delays in trial dates. A larger proportion of adults who may have engaged in services were released at booking, or “force-released” before medication induction or other re-entry services were possible. A careful review of changes in screening, treatment and referral at the population level would more specifically inform strategies to reach a larger proportion of adults with treatment needs cycling through carceral settings.

This small cross-sectional survey of carceral and CTP sites involved in the six-state, prospective EXIT-CJS trial demonstrated that the COVID-19 pandemic affected MOUD service delivery and the capacity for sites to provide re-entry services, including employment and housing support. Telemedicine services were utilized by the majority of carceral and CTP sites to augment MOUD delivery and continue providing healthcare services safely during the pandemic. In addition to novel developments supporting the provision of healthcare services during the COVID-19 pandemic, the development of policies and novel strategies to support individuals during reintegration into the community are critical to promote successful reintegration among persons with OUD.

## Supplementary Information

**Additional file 1.** Supplement 1.

**Additional file 2.** Supplement 1.

## Data Availability

The datasets used and/or analyzed during the current study are available from Dr. Joshua Lee and Ryan McDonald on reasonable request.
