# Supplementary material for: The impact of COVID-19 on the treatment of opioid use disorder in carceral facilities: a cross-sectional study
Source: Health Justice. 2022 Dec 19;10:35. doi: 10.1186/s40352-022-00199-1 (PMC9760540; doi:10.1186/s40352-022-00199-1)
Supplement: Supplementary file 1 — Additional file 1. Supplement 1. [file 40352_2022_199_MOESM1_ESM.docx]

**Section 1. Demographic and organization information**

1. What is your role within your organization?
   - Leadership/Administrative: Security or Operations
   - Leadership/Administrative: Health Services
   - Clinical care provider
   - Behavioral health service provider: Prescriber (e.g., MD, NP, PA)
   - Behavioral health service provider: Not a prescriber
   - Other (Please specify):___________________________________
2. Do you provide direct medical care to patients within your organization/facility?
   - Yes
   - No
3. Is your organization/facility part of a unified corrections system, which integrates state-level prison and jail systems?
   - Yes
   - No
4. *(If Item 3 is “No”)* Please select the structure that best fits your organization/facility:
   - Local jail
   - State prison
   - Other (Please specify):_________________________
5. Are you responding on behalf of a single facility or an organization/correctional system?
   - Single facility
   - Organization/correctional system
6. *(If Item 5 is Organization/Correctional system)* How many facilities are included in your organization/correctional system?

____________ facilities

1. Which model below best describes the health care delivery system in your facility/organization?
   - Direct services model (e.g., healthcare services provided by county or jail employees)
   - Contracted model (e.g., healthcare services provided by contracted vendor/provider)
   - Hybrid model (combination of the two above, e.g., county employed nurses, but contracted physicians)
   - Other (Please specify):___________________________________

**Section 2. Changes in facility/organization population/census**

The next section asks about monthly changes in your organization/facility’s population in the months before (January, February and March 2020) and after (April, May, June, July, and September 2020) the widespread diagnosis of COVID-19 in the United States.

1. In the months before and after COVID-19, what was your average population count (e.g., average daily census per month, or monthly census) across all facilities in your system?

|  | **Population per month across all facilities** | | | | | | | | |
| --- | --- | --- | --- | --- | --- | --- | --- | --- | --- |
|  | Jan 2020 | Feb 2020 | Mar 2020 | Apr 2020 | May 2020 | June 2020 | July 2020 | Aug 2020 | Sept 2020 |
| Population per month |  |  |  |  |  |  |  |  |  |

8b. Please explain how this information was calculated (e.g., average daily census per month, monthly census, other).

________________________________________________________________

1. *(If Item 3 is Yes or Item 4 is Local Jail)* In the months before and after COVID-19, how did the average length of stay (in days) change in your jail facilities for persons who were sentenced? How did the average length of stay change for persons who were not yet sentenced? How did the numbers of new persons admitted who were sentenced and not yet sentenced change?

| Before and after COVID-19, has your facility/organization encountered changes in the: | **Increased** | **No noticeable change** | **Decreased** |
| --- | --- | --- | --- |
| Average length of stay for persons who were sentenced? |  |  |  |
| Number of new admissions per month of persons who were sentenced? |  |  |  |
| Average length of stay for persons who were not sentenced? |  |  |  |
| Number of new admissions per month of persons who were not yet sentenced? |  |  |  |

9b. (*If Item 3 is Yes or Item 4 is State Prison)* In the months before and after COVID-19, how did the average length of stay (in days) change in your prison facilities? How did the number of admissions change?

If possible, please enter the number of persons admitted per month. If you are not able to provide actual numbers at this time, please select “Not available” and comment on the general trends in length of stay and admissions.

*(If data available)*

|  | **Average length of stay in prison facilities**  **(months)** | | | | | | | | |
| --- | --- | --- | --- | --- | --- | --- | --- | --- | --- |
|  | Jan 2020 | Feb 2020 | Mar 2020 | Apr 2020 | May 2020 | June 2020 | July 2020 | Aug 2020 | Sept 2020 |
| Average length of stay |  |  |  |  |  |  |  |  |  |
| Number admitted |  |  |  |  |  |  |  |  |  |

*(If data not available)*

| Before and after COVID-19, has your facility/organization encountered changes in the: | **Increased** | **No noticeable change** | **Decreased** |
| --- | --- | --- | --- |
| Average length of stay |  |  |  |
| Number of persons admitted |  |  |  |

1. In the months before and after COVID-19, how did the number of new admissions change in your facility/organization?

If possible, please enter the number of persons admitted per month. If you are not able to provide actual counts at this time, please select “Not available” and comment on the general trends in new admissions.

*(If data available)*

|  | **Number of persons admitted** | | | | | | | | |
| --- | --- | --- | --- | --- | --- | --- | --- | --- | --- |
|  | Jan 2020 | Feb 2020 | Mar 2020 | Apr 2020 | May 2020 | June 2020 | July 2020 | Aug 2020 | Sept 2020 |
| New admissions |  |  |  |  |  |  |  |  |  |

*(If data not available)*

|  | **Increased** | **No noticeable change** | **Decreased** |
| --- | --- | --- | --- |
| Before and after COVID-19, has your facility/organization had changes in the number of persons admitted per month? |  |  |  |

**Section 3. Screening for opioid use and other substance use disorders**

The following questions ask about screening for opioid and other substance use disorders in your facility/organization.

1. Does your organization/facility have a screening protocol in place to identify people who likely have or may have opioid use problems?
   - Yes
   - No
2. (*If yes to Item 12)* Since COVID-19, has your facility had any changes in:

|  | **Increased** | **No noticeable change** | **Decreased** |
| --- | --- | --- | --- |
| The number of persons screened for opioid use disorder? |  |  |  |

1. In the months before and after COVID-19, did your facility/organization have any changes in the number of of the persons admitted to your facility/organization, who:

|  | **Increased** | **No noticeable change** | **Decreased** |
| --- | --- | --- | --- |
| Had recently used opioids based on self-report, referral, or urine testing? |  |  |  |
| Needed opioid withdrawal management? |  |  |  |
| Were screened using a screener or questionnaire to determine possible opioid use disorder? |  |  |  |

**Section 4. Treatment for opioid use and other substance use disorders**

The next section asks about the treatment of opioid use disorders (OUD) and other substance use disorders in your facility/organization.

Several questions ask about use of medication for opioid use disorder (MOUD), which can include:

- Methadone: This is the full agonist medication used for the treatment of OUD. Brand names include Methadose®, Dolophine®, Diskets®, and Methadone Intensol®.
- Buprenorphine: This is any partial agonist buprenorphine/buprenorphine-naloxone medication used for the treatment of OUD. Brand names include Suboxone®, Subutex®, Sublocade®, Zubsolv®, Bunavail®, Butrans®, Buprenex®, Probuphine®, and Belbuca®.
- Naltrexone: This is a full antagonist medication used for the treatment of OUD. Brand names include Vivitrol®, ReVia®, Adepend®, Depade®, Nalorex®, and Trexan®.

1. Does your facility/organization prescribe medication for opioid use disorder (MOUD) to treat opioid use disorder (OUD) for incarcerated/detained persons?

This could be prescribed either by corrections medical staff or through a health services or community partner working within the facility.

- - Yes
  - No

1. *(If yes to Item 15)* For what purpose is MOUD prescribed in your facility?

*Select all that apply*

- - Opioid withdrawal management
  - Maintenance for any persons with opioid use disorder
  - Induction and maintenance only immediately prior to release
  - Maintenance only for pregnant women
  - Maintenance only for persons entering on MOUD
  - Other (Please specify):________________________________

1. (*If yes to Item 15)* Which medications for opioid use disorder are available to incarcerated/detained persons within your facility/organization?
   - Methadone
   - Oral buprenorphine
   - Extended-release buprenorphine
   - Oral buprenorphine/naloxone
   - Extended-release naltrexone
2. *(If “Methadone” endorsed as response to Item 17)* Is your facility/organization certified as an opioid treatment program?
   - Yes
   - No
3. *(If no to item 18)* How is methadone dosed to incarcerated/detained persons in your facility/organization?

*Select all that apply.*

- - Onsite by an external agency/provider
  - Offsite by medical transport
  - Offsite by a mobile unit
  - Other (Please specify):________________________________________

1. *(If “Methadone” endorsed as response to Item 17)* In the months before and after COVID-19, has your facility/organization enacted any changes to methadone dosing procedures?
   - Yes
   - No
2. *(If “Methadone” endorsed as response to Item 17 and Items 19 and 20 indicate dosing occurring onsite)* Since COVID-19, what changes in prescribing or dosing procedures for methadone did your facility/organization enact?

*Select all that apply.*

- - Enhanced cleaning regimens (e.g., cleaning surfaces in dosing space with EPA-approved hand sanitizer multiple times per day)
  - Altered dosing location (e.g., delivering methadone to a patient’s living space or cell, dosing patients in a separate room if they present with a fever or cough)
  - Provision of cloth masks to patients
  - Implementing cohort dosing
  - Screening patients for COVID-19 symptoms prior to dosing
  - Limiting access or enhancing screening of external agency staff/providers (e.g., screening external agency/providers for COVID-19 before allowing entry to the facility, moving visits with external agency/providers to telehealth visits
  - Other (Please specify):__________________________________

1. *(If “Methadone” endorsed as response to Item 17 and Items 19 and 20 indicate dosing occurring offsite by medical transport )* Since COVID-19, what changes in prescribing or dosing procedures for methadone did your facility/organization enact?

*Select all that apply.*

- - Enhanced cleaning regimens (e.g., cleaning surfaces in transport vehicle with EPA-approved hand sanitizer multiple times per day)
  - Altered dosing location (e.g., allowing community partner to deliver methadone to a patient’s living space or cell)
  - Provision of cloth masks to patients
  - Implementing cohort dosing
  - Screening patients for COVID-19 symptoms prior to transport
  - Eliminating or pausing offsite transport of patients to community organization for dosing
  - Other (Please specify):__________________________________

1. *(If “Methadone” endorsed as response to Item 17 and Items 19 and 20 indicate dosing occurring offsite by mobile unit )* Since COVID-19, what changes in prescribing or dosing procedures for methadone did your facility/organization enact?

*Select all that apply.*

- - Enhanced cleaning regimens (e.g., cleaning surfaces in transport vehicle with EPA-approved hand sanitizer multiple times per day)
  - Altered dosing location (e.g., allowing the mobile unit to deliver methadone to a patient’s living space or cell)
  - Provision of cloth masks to patients
  - Implementing cohort dosing
  - Screening patients for COVID-19 symptoms prior to transport
  - Eliminating or pausing offsite transport of patients to the mobile unit for dosing
  - Other (Please specify):__________________________________

1. *(If yes to Item 15)* In the months before and after COVID-19, how many incarcerated/detained persons were receiving MOUD for any reason (e.g., withdrawal management, maintenance) in your facility/organization?

|  | **Number of incarcerated/detained persons receiving medication for opioid use disorder (MOUD)**  **(Total number per month)** | | | | | | | | |
| --- | --- | --- | --- | --- | --- | --- | --- | --- | --- |
|  | Jan 2020 | Feb 2020 | Mar 2020 | Apr 2020 | May 2020 | June 2020 | July 2020 | Aug 2020 | Sept 2020 |
| Total receiving MOUD |  |  |  |  |  |  |  |  |  |

1. *(If yes to any medications listed in Item 18)* Of those receiving MOUD for maintenance, how did the number of persons receiving methadone, buprenorphine, buprenorphine/naloxone, or naltrexone in your facility/organization change in the months before and after COVID-19?

| In your facility/organization, how did the number of prescriptions change in the months before and after COVID-19 for: | **Increased** | **No noticeable change** | **Decreased** |
| --- | --- | --- | --- |
| Methadone |  |  |  |
| Sublingual buprenorphine (e.g., Suboxone, Subutex) |  |  |  |
| Extended-release buprenorphine (e.g., Sublocade, Brixadi, Probuphine) |  |  |  |
| Extended-release naltrexone (e.g., Vivitrol) |  |  |  |
| Other (Please specify):_____ |  |  |  |

1. Does your facility/organization have any onsite providers (e.g., Physicians, Nurse Practitioners, Physicians Assistants, Clinical Nurse Specialists, Certified Registered Nurse Anesthetists, or Certified Nurse-Midwifes) who are waivered to prescribed buprenorphine?
   - Yes
   - No
2. *(If yes to Item 26)* In the months before and after COVID-19, did your facility/organization have any:

|  | **Increased** | **No noticeable change** | **Decreased** |
| --- | --- | --- | --- |
| Changes in the number of providers waivered to prescribe buprenorphine? |  |  |  |

1. *(If yes to Item 15 and Buprenorphine or Naltrexone option endorsed on Item 17)* In the months before and after COVID-19, did your facility encounter or enact any changes in dosing procedures for buprenorphine/naloxone?
   - Yes
   - No
2. *(If yes to Item 28)* In the months before and after COVID-19, what changes in prescribing or dosing procedures for buprenorphine and naloxone did your facility/organization enact?

*Select all that apply.*

- - Enhanced cleaning regimens (e.g., cleaning all surfaces in the medical unit several times each day with EPA-approved sanitizers)
  - Altered dosing location (e.g., delivering doses to a patient’s living space or cell)
  - Provision of cloth masks to patients
  - Implementing cohort dosing
  - Screening patients for COVID-19 symptoms prior to dosing
  - Reducing the number of visits for dosing (e.g., moving visits with providers for prescriptions to telehealth visits, shifting patients to long-acting formulations to reduce dosing visits)
  - Other (Please specify):__________________________________

1. *(If yes to Items 20 or Item 28)* Have changes in MOUD dosing protocols resulted in any changes to protocols seeking to prevent medication diversion?
   - Yes
   - No
2. In the months before and after COVID-19, did your organization/facility have any changes in the number of incarcerated/detained persons initiating opioid withdrawal management protocols for opioid use disorder?

|  | **Increased** | **No noticeable change** | **Decreased** |
| --- | --- | --- | --- |
| Number of persons initiating opioid withdrawal management? |  |  |  |

1. In the months before and after COVID-19 among those who initiated opioid withdrawal management, did your organization/facility have any changes in the number of incarcerated/detained persons who received MOUD (e.g., a taper), versus other prescription medications only, versus no medication or over-the-counter medications only for opioid withdrawal management?

| Number of persons initiating opioid withdrawal management who received: | **Increased** | **No noticeable change** | **Decreased** |
| --- | --- | --- | --- |
| MOUD |  |  |  |
| Other prescription medications |  |  |  |
| No medication or over-the-counter medications only |  |  |  |

1. In the months before and after COVID-19, how did the number of incarcerated/detained persons entering on medication for opioid use disorder (MOUD) change within your facility/organization? How did the number of these persons continued on MOUD in your facility/organization change?

| Number of persons: | **Increased** | **No noticeable change** | **Decreased** |
| --- | --- | --- | --- |
| Entering the facility on MOUD (prescribed prior to booking) |  |  |  |
| Continued on MOUD |  |  |  |
| Tapered off MOUD prior to release |  |  |  |

1. *(If yes to Item 15)* In the months before and after COVID-19, how did the number of incarcerated/detained persons being inducted onto MOUD in your facility/organization change?

| Number of persons: | **Increased** | **No noticeable change** | **Decreased** |
| --- | --- | --- | --- |
| Inducted on MOUD (Total) |  |  |  |
| Started for withdrawal management only |  |  |  |
| Started for maintenance |  |  |  |

1. In the months before and after COVID-19, did your organization have changes in the number of incarcerated/detained persons released:

- On MOUD without a referral to community treatment

- Not on MOUD but with referral to community treatment for MOUD

- On MOUD and with referral to community treatment?

| Changes in the number of persons: | **Increased** | **No noticeable change** | **Decreased** |
| --- | --- | --- | --- |
| Released on MOUD without a referral? |  |  |  |
| Released not on MOUD, but with referral to community treatment for MOUD? |  |  |  |
| Released on MOUD and referred to community treatment? |  |  |  |

1. *(If yes to Item 15)* As a result of COVID-19, did your facility/organization’s MOUD program stop?
   - Yes
   - No
2. *(If yes to Item 15)* As a result of COVID-19, did your facility/organization’s MOUD program have reduced capacity?
   - Yes
   - No
3. (*If yes to Item 15)* Since COVID-19, has your facility had any changes in:

|  | **Increased** | **No noticeable change** | **Decreased** |
| --- | --- | --- | --- |
| The demand for MOUD? |  |  |  |

1. What types of non-medication treatments are available to incarcerated/detained persons with substance use disorders in your facility/organization?

*Select all that apply*

- - Outpatient substance use treatment by licensed provider (8 hours of fewer a week)
  - Intensive outpatient substance use treatment by licensed provider (9 or more hours per week)
  - Therapeutic community within correctional setting by licensed provider
  - Other recovery-based unit within correctional setting
  - Transfer to residential treatment or inpatient substance use treatment program in the community by licensed provider
  - Onsite short-term or long-term residential substance use treatment by licensed provider
  - Co-occurring substance use and mental health services by licensed provider
  - Mutual- or self-help group meetings (e.g. Alcoholics Anonymous, Narcotics Anonymous, SMART Recovery)
  - Peer mentor/navigator/recovery coach
  - Other (Please specify):___________________________

1. *(Options will reflect selections from Item 39)* Since COVID-19, has your facility had any changes in the provision of:

|  | **Increased** | **No noticeable change** | **Decreased** |
| --- | --- | --- | --- |
| Outpatient treatment onsite? |  |  |  |
| Intensive outpatient treatment onsite? |  |  |  |
| Treatment within an onsite therapeutic community? |  |  |  |
| Treatment within an onsite recovery-based unit? |  |  |  |
| Transfers to community residential or inpatient treatment? |  |  |  |
| Onsite residential or inpatient treatment? |  |  |  |
| Co-occurring substance use and mental health treatment? |  |  |  |
| Mutual- or self-help group meetings? |  |  |  |
| Peer mentor/navigator/recovery coach services? |  |  |  |

1. Prior to COVID-19, did your facility/organization use telemedicine to provide treatment for any health conditions?
   - Yes
   - No
   - Not sure
2. (*If yes to Item 41*): Prior to COVID-19, did your facility/organization use telemedicine to induct incarcerated/detained persons onto MOUD (e.g., buprenorphine, methadone, naltrexone)?
   - Yes
   - No
   - Not sure
3. (*If yes to Item 41*): Prior to COVID-19, did your facility/organization use telemedicine appointments for incarcerated/detained persons maintained on MOUD?
   - Yes
   - No
   - Not sure
4. (If yes to Item 41): Since COVID-19, has your facility had any changes in:

|  | **Increased** | **No noticeable change** | **Decreased** |
| --- | --- | --- | --- |
| Use of telemedicine for all health conditions? |  |  |  |
| Use of telemedicine for MOUD? |  |  |  |

1. Which of the following technology/methods are being used by incarcerated/detained persons within your facility/organization to participate in substance use treatment or recovery support?

*Select all that apply.*

- - Computers
  - Tablets
  - Portable kiosks
  - Kiosks
  - Cell phones
  - Internet
  - Text messaging
  - Email
  - Video calls
  - Other applications or software programs (Please specify):_____________

1. *(Options will reflect selections from Item 45)* Since COVID-19, how has your facility/organization’s use of the following technology programs for substance use treatment or recovery support changed?

|  | **Increased** | **No noticeable change** | **Decreased** |
| --- | --- | --- | --- |
| Computers |  |  |  |
| Tablets |  |  |  |
| Portable kiosks |  |  |  |
| Kiosks |  |  |  |
| Cell phones |  |  |  |
| Internet |  |  |  |
| Text messaging |  |  |  |
| Email |  |  |  |
| Video calls |  |  |  |
| Other applications or software programs |  |  |  |

**Section 5. Other organizational policies and COVID-19**

1. Has your organization/facility enacted or encountered any changing policies due to COVID-19?
   - Yes
   - No
   - Not sure
2. *(If yes to Item 47)* Which changing policies has your facility/organization encountered or enacted due to COVID-19?

*Select all that apply*

- - Reduction of the state prison population
  - Reduction of jail population
  - Reduction of jail entries or admissions
  - Reduction of the state prison entries or admissions
  - Reduction of face-to-face medical visits
  - Elimination of medical co-pays
  - Reduction of face-to-face visitation of inmates
  - Changes in movement policies
  - Other (Please specify):_________________________

**Section 6. Release planning**

1. During the month prior to release, do staff in your facility/organization:

|  | **Yes** | **No** |
| --- | --- | --- |
| Induct (start) incarcerated/detained persons on MOUD? |  |  |
| Schedule appointments with MOUD providers in the community? |  |  |
| Provide assistance completing intake paperwork for quick access to MOUD providers in the community? |  |  |
| Facilitate exchange of key information with the MOUD provider in the community? |  |  |
| Provide incarcerated/detained persons with the name(s) of MOUD providers in their community of release? |  |  |
| Coordinate MOUD services with parole/probation? |  |  |
| Assist with reactivating and/or applying for Medicaid, Veterans benefits or other types of insurance for payment of MOUD? |  |  |
| Connect incarnated/detained persons to a peer mentor/navigator/recovery coach? |  |  |
| Provide or arrange transportation to MOUD provider in the community? |  |  |
| Provide a bridge supply of multiple doses/days of MOUD? |  |  |
| Provide a written prescription for MOUD? |  |  |
| Engage in other activities to facilitate linkage to MOUD? (Please specify):___________________ |  |  |

1. *(Options will reflect selections from Item 49)* In the month prior to release, how has COVID-19 impacted your facility/organization’s ability to:

|  | **Less difficult** | | **No noticeable change** | | **More difficult** |
| --- | --- | --- | --- | --- | --- |
| Induct (start) incarcerated/detained persons on MOUD? |  | |  | |  |
| Schedule appointments with MOUD providers in the community? |  | |  | |  |
| Provide assistance completing intake paperwork for quick access to MOUD providers in the community? |  | |  | |  |
| Facilitate exchange of key information with the MOUD provider in the community? |  | |  | |  |
| Provide incarcerated/detained persons with the name(s) of MOUD providers in their community of release? |  | |  | |  |
| Coordinate MOUD services with parole/probation? |  | |  | |  |
| Assist with reactivating and/or applying for Medicaid, Veterans benefits or other types of insurance for payment of MOUD? |  | |  | |  |
| Connect incarnated/detained persons to a peer mentor/navigator/recovery coach? |  | |  | |  |
| Provide or arrange transportation to MOUD provider in the community? |  | |  | |  |
| Provide a bridge supply of multiple doses/days of MOUD? |  |  | |  | |
| Provide a written prescription for MOUD? |  |  | |  | |
| Engage in other activities to facilitate linkage to MOUD? (Please specify):___________________ |  |  | |  | |

1. Does your facility/organization provide naloxone kits at release to incarcerated/detained persons?
   - Yes
   - No
2. *(If yes to Item 51)* Naloxone kits are provided to-
   - Everyone
   - Only individuals with opioid use problems or opioid use disorder
   - Other (Please specify):_________________________________
3. Since COVID-19, how has your facility/organization’s provision of naloxone kits at release changed?

|  | **Increased** | **No noticeable change** | **Decreased** |
| --- | --- | --- | --- |
| Provision of naloxone kits at release |  |  |  |

1. Since COVID-19, are there any processes related to quarantining or contact tracing that have changed protocols for obtaining housing for incarcerated/detained persons post-release?
   - Yes (Please specify):__________________________________
   - No
2. Post-release, how has COVID-19 impacted your facility/organization’s ability to:

|  | **Less difficult** | **No noticeable change** | **More difficult** |
| --- | --- | --- | --- |
| Locate housing for incarcerated/detained persons in the community? |  |  |  |
| Help incarcerated/detained persons find employment in the community? |  |  |  |
| Provide transportation (e.g., bus cards/tokens) home for incarcerated/detained persons? |  |  |  |

56. Thinking about the changes your organization/facility has already implemented due to COVID-19, do you anticipate further changes throughout the rest of 2020? If so, please describe these anticipated changes below.

___________________________________________________________
