# Supplementary material for: The impact of COVID-19 on the treatment of opioid use disorder in carceral facilities: a cross-sectional study
Source: Health Justice. 2022 Dec 19;10:35. doi: 10.1186/s40352-022-00199-1 (PMC9760540; doi:10.1186/s40352-022-00199-1)
Supplement: Supplementary file 2 — Additional file 2. Supplement 1. [file 40352_2022_199_MOESM2_ESM.docx]

**EXIT-CJS Community Program COVID-19 Survey**

**Section 1. Demographic and organization information**

1. How would you describe your organization?
   - Tertiary care medical center
   - Stand-alone outpatient medical and/or behavioral health center
   - Licensed addiction treatment provider
   - Opioid treatment program (OTP)
   - Other (Please specify):_______________________________
2. What is your role within your organization?
   - Leadership/Administrative: CEO, Medical Director
   - Leadership/Administrative: Administrator
   - Clinical care provider: Prescriber (e.g., MD, NP, PA)
   - Counselor or health service provider: Not a prescriber, counselor, case manager (MSW, MA, MPH)
   - Other (Please specify):___________________________________
3. Are you a treatment provider (vs. administration only) who provides direct medical care to patients within your organization/facility?
   - Yes
   - No

4. Do you have specific contracts or treatment tracks for criminal justice-involved (post-release, parole, drug court, etc) adults with opioid use disorders (OUD)?

- Yes
- No

4b. If Yes, please describe: _______________________________________________

____________________________________________________________________________________________________________________________________________

5. What types of non-medication treatments are available to CJS-involved persons (post-release, parole, drug court, etc) with substance use disorders in your facility/organization?

*Select all that apply*

- - Outpatient substance use treatment by licensed provider (8 hours or fewer a week)
  - Intensive outpatient substance use treatment by licensed provider (9 or more hours per week)
  - Therapeutic community within correctional setting by licensed provider
  - Other recovery-based unit within correctional setting
  - Transfer to residential treatment or inpatient substance use treatment program in the community by licensed provider
  - Onsite short-term or long-term residential substance use treatment by licensed provider
  - Co-occurring substance use and mental health services by licensed provider
  - Mutual- or self-help group meetings (e.g. Alcoholics Anonymous, Narcotics Anonymous, SMART Recovery)
  - Peer mentor/navigator/recovery coach
  - Other (Please specify):___

Several questions ask about use of medication for opioid use disorder (MOUD), which can include:

- Methadone: This is the full agonist medication used for the treatment of OUD. Brand names include Methadose®, Dolophine®, Diskets®, and Methadone Intensol®.
- Buprenorphine: This is any partial agonist buprenorphine/buprenorphine-naloxone medication used for the treatment of OUD. Brand names include Suboxone®, Subutex®, Sublocade®, Zubsolv®, Bunavail®, Butrans®, Buprenex®, Probuphine®, and Belbuca®.
- Naltrexone: This is a full antagonist medication used for the treatment of OUD. Brand names include Vivitrol®, ReVia®, Adepend®, Depade®, Nalorex®, and Trexan®.

1. For what purpose does your organization prescribe medications for opioid use disorders (MOUD)?

*Select all that apply*

- - Opioid withdrawal management
  - Maintenance for adults with opioid use disorder
  - Maintenance for pregnant women
  - Maintenance for juveniles (younger than 18 yo)
  - Other (Please specify):________________________________

1. Which medications for opioid use disorder (MOUD) are available?

*Select all that apply*

- - Methadone
  - Oral sublingual buprenorphine (Subutex)
  - Oral sublingual buprenorphine/naloxone (Suboxone, Zubsolv, generics)
  - Extended-release buprenorphine (Sublocade)
  - Extended-release naltrexone (Vivitrol)
  - Oral naltrexone

1. Does your facility/organization have any onsite providers (e.g., Physicians, Nurse Practitioners, Physicians Assistants, Clinical Nurse Specialists, Certified Registered Nurse Anesthetists, or Certified Nurse-Midwifes) who are waivered to prescribed buprenorphine?
   - Yes
   - No

**Section 2. Changes in facility/organization population/census**

The next section asks about monthly changes in your organization/facility’s population in the months before (Jan-Mar 2020) and after (April-present 2020) the widespread diagnosis of COVID-19 in the United States.

1. In the months before and after COVID-19, what was your average OUD (opioid use disorder) patient population count (e.g., average daily census per month, or monthly census) across all clinics and/or parts of your system?

|  | **Population per month across your organization** | |
| --- | --- | --- |
|  | Jan-Mar 2020 | Apr-Present 2020 |
| Population per month |  |  |

*9b.* Please explain how this information was calculated (e.g., average daily census per month, monthly census, other).

________________________________________________________________

1. How has the length of stay in OUD treatment in your program changed during COVID-19?

| Before and after COVID-19, has your facility/organization encountered changes in the: | **Increased** | **No noticeable change** | **Decreased** |
| --- | --- | --- | --- |
| Average length of stay? |  |  |  |
| Admissions from CJS referral sources? |  |  |  |
| Length of stay for CJS referred sources? |  |  |  |

**Section 3. Screening, Admissions, and Treatments for OUD and other drug and alcohol use disorders**

The following questions ask about screening and admissions for opioid and other substance use disorders in your facility/organization.

11. Since COVID-19, have your OUD clinical program(s) had any changes in:

|  | **Increased** | **No noticeable change** | **Decreased** |
| --- | --- | --- | --- |
| The number of persons admitted for opioid use disorder? |  |  |  |
| The % of persons admitted for OUD vs. other use disorders |  |  |  |
| The number of persons admitted who needed opioid withdrawal management? |  |  |  |
| The number of persons admitted who self-reported or screened positive (urine) for fentanyl or related analogues of fentanyl? |  |  |  |

1. In the months before and after COVID-19, how many persons were receiving MOUD for any reason (e.g., withdrawal management, maintenance) in your facility/organization?

|  | **Number of individual patients receiving medication for opioid use disorder (MOUD)**  **(Total number per month)** | |
| --- | --- | --- |
|  | Jan-Mar 2020 | April-Present 2020 |
| Total receiving MOUD per month |  |  |

1. Of those receiving MOUD for maintenance, how did the number of persons receiving methadone, buprenorphine, buprenorphine/naloxone, or naltrexone in your facility/organization change in the months before and after COVID-19?

| In your facility/organization, how did the number of prescriptions change in the months before and after COVID-19 for: | **Increased** | **No noticeable change** | **Decreased** |
| --- | --- | --- | --- |
| Methadone |  |  |  |
| Sublingual buprenorphine (e.g., Suboxone, Subutex, generics) |  |  |  |
| Extended-release buprenorphine (e.g., Sublocade, Brixadi, Probuphine) |  |  |  |
| Extended-release naltrexone (e.g., Vivitrol) |  |  |  |
| Other (Please specify):_____ |  |  |  |

1. In the months before and after COVID-19, did your program have any:

|  | **Increased** | **No noticeable change** | **Decreased** |
| --- | --- | --- | --- |
| Changes in the number of providers waivered to prescribe buprenorphine? |  |  |  |

1. In the months before and after COVID-19, did your facility encounter or enact any changes in dosing procedures for buprenorphine/naloxone?
   - Yes
   - No
2. *(If yes to Item 15)* In the months before and after COVID-19, what changes in prescribing or dosing procedures for buprenorphine/naloxone did your facility/organization enact?

*Select all that apply.*

- - Enhanced cleaning regimens (e.g., cleaning all surfaces in the medical unit several times each day with EPA-approved sanitizers)
  - Altered dosing location (e.g., delivering doses to a patient’s living space or cell)
  - Provision of cloth masks to patients
  - Implementing cohort dosing
  - Screening patients for COVID-19 symptoms prior to dosing
  - Reducing the number of visits for dosing (e.g., moving visits with providers for prescriptions to telehealth visits, shifting patients to long-acting formulations to reduce dosing visits)
  - Other (Please specify):__________________________________

1. *(If yes to Items 15 and endorsed on item 16)* Have changes in MOUD dosing protocols resulted in any changes to protocols seeking to prevent medication diversion?
   - Yes
   - No
2. In the months before and after COVID-19, how did the number of CJS-involved (post-release, parole, drug court, etc) persons being inducted onto MOUD in your facility/organization change?

| Number of persons: | **Increased** | **No noticeable change** | **Decreased** |
| --- | --- | --- | --- |
| Inducted on MOUD (Total) |  |  |  |
| Started for withdrawal management only |  |  |  |
| Started for maintenance |  |  |  |

1. As a result of COVID-19, did any of your facility/organization’s MOUD program stop?
   - Yes
   - No

19b. If yes, Please describe: ____________________________________________________________________________________________________________________________________________

1. Since COVID-19, has your facility had any changes in:

|  | **Increased** | **No noticeable change** | **Decreased** |
| --- | --- | --- | --- |
| The demand for MOUD? |  |  |  |
| Capacity? |  |  |  |

1. Since COVID-19, has your facility had any changes in the provision of:

|  | **Increased** | **No noticeable change** | **Decreased** |
| --- | --- | --- | --- |
| Outpatient treatment onsite? |  |  |  |
| Intensive outpatient treatment onsite? |  |  |  |
| Treatment within an onsite therapeutic community? |  |  |  |
| Treatment within an onsite recovery-based unit? |  |  |  |
| Transfers to community residential or inpatient treatment? |  |  |  |
| Onsite residential or inpatient treatment? |  |  |  |
| Co-occurring substance use and mental health treatment? |  |  |  |
| Mutual- or self-help group meetings? |  |  |  |
| Peer mentor/navigator/recovery coach services? |  |  |  |

1. Prior to COVID-19, did your facility/organization use telemedicine to provide treatment for any health conditions?
   - Yes
   - No
   - Not sure
2. Prior to COVID-19, did your facility/organization use telemedicine to induct onto MOUD (e.g., buprenorphine, methadone, naltrexone)?
   - Yes
   - No
   - Not sure
3. Prior to COVID-19, did your facility/organization use telemedicine appointments for persons maintained on MOUD?
   - Yes
   - No
   - Not sure
4. Since COVID-19, has your facility had any changes in:

|  | **Increased** | **No noticeable change** | **Decreased** |
| --- | --- | --- | --- |
| Use of telemedicine for all health conditions? |  |  |  |
| Use of telemedicine for MOUD? |  |  |  |

1. Which of the following technology/methods are being used by OUD patients in your program(s) to participate in substance use treatment or recovery support?

*Select all that apply.*

- - Computers
  - Tablets
  - Portable kiosks
  - Kiosks
  - Cell phones
  - Internet
  - Text messaging
  - Email
  - Video calls
  - Other applications or software programs (Please specify):_____________

1. *(Options will reflect selections from Item 26)* Since COVID-19, how has your facility/organization’s use of the following technology programs for substance use treatment or recovery support changed?

|  | **Increased** | **No noticeable change** | **Decreased** |
| --- | --- | --- | --- |
| Computers |  |  |  |
| Tablets |  |  |  |
| Portable kiosks |  |  |  |
| Kiosks |  |  |  |
| Cell phones |  |  |  |
| Internet |  |  |  |
| Text messaging |  |  |  |
| Email |  |  |  |
| Video calls |  |  |  |
| Other applications or software programs |  |  |  |

1. *(If your program is a methadone/opioid treatment program [Item 7])* In the months before and after COVID-19, has your facility/organization enacted any changes to methadone dosing procedures?
   - Yes
   - No

28b. *If Yes to Item 28,* Since COVID-19, what changes in prescribing or dosing procedures for methadone did your facility/organization enact?

*Select all that apply.*

- - Enhanced cleaning regimens (e.g., cleaning surfaces in dosing space with EPA-approved hand sanitizer multiple times per day)
  - Altered dosing location (e.g., delivering methadone to a patient’s living space or cell, dosing patients in a separate room if they present with a fever or cough)
  - Provision of cloth masks to patients
  - Implementing cohort dosing
  - Screening patients for COVID-19 symptoms prior to dosing
  - Limiting access or enhancing screening of external agency staff/providers (e.g., screening external agency/providers for COVID-19 before allowing entry to the facility, moving visits with external agency/providers to telehealth visits
  - Other (Please specify):__________________________________

**Section 5. Other organizational policies and COVID-19**

1. Has your organization/facility enacted or encountered any changing policies due to COVID-19?
   - Yes
   - No
   - Not sure
2. *(If yes to Item 29)* Which changing policies has your facility/organization encountered or enacted due to COVID-19?

*Select all that apply*

Reductions in new admissions (new patient treatment appointments)

- - Reduction of face-to-face medical visits
  - Elimination of medical co-pays
  - Other (Please specify):_________________________

1. Does your facility/organization provide naloxone kits?
   - Yes
   - No
2. *(If yes to Item 31)* Naloxone kits are provided to-
   - Everyone
   - Only individuals with opioid use problems or opioid use disorder
   - Other (Please specify):_________________________________
3. Since COVID-19, how has your facility/organization’s provision of naloxone kits at release changed?

|  | **Increased** | **No noticeable change** | **Decreased** |
| --- | --- | --- | --- |
| Provision of naloxone kits |  |  |  |

1. Since COVID-19, are there any processes related to quarantining or contact tracing that have changed protocols for housing supports or homeless services?
   - Yes (Please specify):__________________________________
   - No
2. Post-release, how has COVID-19 impacted your facility/organization’s ability to:

|  | **Less difficult** | **No noticeable change** | **More difficult** |
| --- | --- | --- | --- |
| Housing assistance and homeless supports? |  |  |  |
| Employment programs and supports? |  |  |  |
| Transportation support (e.g., fare cards, Uber credits)? |  |  |  |

42. Thinking about the changes your organization/facility has already implemented due to COVID-19, do you anticipate further changes throughout the rest of 2020? If so, please describe these anticipated changes below.

________________________________________________________________________________________________________________________________________________________________________________________________________________________________________________________________________________________
